# Inavolisib-induced fulminant-like diabetes and hyperosmolar hyperglycemic state: a case report

**DOI:** 10.3389/fendo.2026.1747317

**Published:** 2026-05-08

**Authors:** Hongyu Li, Chenxiang Cao, Lixia Jin, Jianzhong Xiao, Wenhui Zhao, Xiaojing Wang

**Affiliations:** Department of Endocrinology, Beijing Tsinghua Changgung Hospital, School of Clinical Medicine, Tsinghua Medicine, Tsinghua University, Beijing, China

**Keywords:** breast cancer, fulminant diabetes, hyperosmolar hyperglycemic state, inavolisib, PI3K inhibitor

## Abstract

**Background:**

Inavolisib, a recently approved phosphatidylinositol 3-kinase α (PI3Kα) inhibitor for advanced breast cancer, carries a notable risk of hyperglycemia. Despite this established, on-target adverse effect, clinical endocrinologists often lack sufficient awareness regarding both the risk profile and the specific management strategies required for Inavolisib-induced hyperglycemia. The objective of this report is to describe a patient with fulminant-like diabetes accompanied by Hyperosmolar Hyperglycemic State (HHS) following Inavolisib administration.

**Case presentation:**

A 59-year-old female patient with metastatic breast cancer developed rapid-onset and severe hyperglycemia (blood glucose: 48.0 mmol/L at emergency presentation) progressing to HHS within 72 hours of initiating Inavolisib therapy. She presented with fatigue but showed no evidence of ketoacidosis. She had no personal or family history of diabetes mellitus and multiple prior random blood glucose measurements were within normal range. Admission laboratory findings included glycated hemoglobin A1c (HbA1c) 5.7% (reference: 4.0-6.0%), fasting plasma glucose 8.6 mmol/L, fasting insulin 41.5 μU/mL (reference: 2.6–24.9 μU/mL), and fasting C-peptide 10.2 ng/mL (reference: 1.1–4.4 ng/mL), Diabetes-related autoantibodies (ICA, GADA, IAA) were negative. After discontinuation of Inavolisib and administration of intensive insulin therapy, the patient’s hyperglycemia resolved rapidly.

**Conclusion:**

Inavolisib-induced hyperglycemia may occur abruptly after treatment initiation and can rapidly progress to severe hyperglycemic emergencies such as HHS, even in patients without known dysglycemia. Normal baseline glycemic indices do not reliably exclude the risk of severe toxicity. Early metabolic assessment, close glucose monitoring, prompt interruption of inavolisib when clinically indicated, and rapid insulin-based management are essential for timely recognition and safe clinical care.

## Introduction

Phosphatidylinositol 3-kinase α (PI3Kα) inhibitors represent a crucial class of agents for managing PIK3CA-mutated, hormone receptor-positive/human epidermal growth factor receptor 2-negative (HR+/HER2-) advanced breast cancer (1–4). Inavolisib, a novel, potent, and highly selective PI3Kα inhibitor, has recently been approved for marketing (5), providing a new and effective therapeutic avenue (6, 7). However, the confirmed clinical efficacy is intrinsically linked to a pronounced metabolic liability: hyperglycemia stands out as one of the most common and clinically notable adverse events (7, 8). This toxicity arises directly from the drug’s mechanism of action, involving the inhibition of the PI3Kα-AKT pathway—a key node in insulin signaling—which precipitates severe insulin resistance and diminished glucose uptake (9).

As a newly marketed drug with a limited clinical history, Inavolisib’s package insert lists hyperglycemia as an established adverse effect (8). However, despite this warning, clinical endocrinologists may currently lack sufficient specialized awareness regarding the agent’s unique metabolic profile, including the rapid onset of hyperglycemia and the potential for severe disease progression. This gap in knowledge may impede the timely and standardized clinical response. The objective of this report is to describe a patient with rapid-onset and severe hyperglycemia following the administration of Inavolisib, aims to alert clinicians to the risk of fulminant-like diabetes and Hyperosmolar Hyperglycemic State (HHS) induced by Inavolisib and share relevant management experience.

## Case report

A 59-year-old female patient was admitted to the Department of Endocrinology following a two-day history of newly detected hyperglycemia. Her main symptoms were fatigue accompanied by mild chest tightness, without typical manifestations of polydipsia, polyphagia, or polyuria. One day prior to her inpatient admission, she had presented to the emergency department due to progressively worsening fatigue. On physical examination in the emergency department, the findings were as follows: body temperature 36.2°C; pulse 99 beats per minute; respiratory rate 16 breaths per minute; blood pressure 105/58 mmHg; oxygen saturation 96%; body weight 53.55 kg; height 165.5 cm; body mass index (BMI) 19.55 kg/m²; waist circumference 72 cm. The patient was in generally good condition, with clear consciousness; bilateral pupils were equal in size and round, with sensitive light reflexes. Chest and abdominal examinations were negative; no pitting edema was noted in the lower extremities. Laboratory tests revealed severe hyperglycemia and hyperosmolality: arterial blood glucose 48.0 mmol/L; arterial blood sodium 122 mmol/L (corrected sodium: 134 mmol/L; reference: 135–145 mmol/L); arterial blood potassium 5.5 mmol/L (reference: 3.5-5.3 mmol/L); arterial blood pH 7.43; bicarbonate 22.6 mmol/L (reference: 22–28 mmol/L), lactic acid 2.0 mmol/L; effective serum osmotic pressure 327 mOsm/L (reference: 275–295 mOsm/kg) and urine ketone bodies negative. After fluid replacement and intravenous insulin pump therapy in the emergency department, her blood glucose level decreased. The random blood glucose at admission was 13.9 mmol/L (reference: 3.9-6.1 mmol/L). The patient denied any history of prediabetes or gestational diabetes. Her body mass index (BMI) was 19.55 kg/m², and she reported a fair appetite with no significant recent weight changes, no major recent lifestyle change, and no evident acute psychosocial stressor before presentation. She had no history of hypercholesterolemia and denied any prior use of glucocorticoids. Historical random blood glucose measurements ranged from 5.5 to 5.9 mmol/L between 2023 and 2024. An out-of-hospital metabolic panel from November 2024 showed a fasting glucose of 5.8 mmol/L (reference: 3.9–6.1 mmol/L), fasting insulin of 8.5 μU/mL (reference: 2.6–24.9 μU/mL), and C-peptide of 3.2 ng/mL (reference: 1.1–4.4 ng/mL)(calculated HOMA-IR: 2.2). Upon admission, her glycated hemoglobin A1c (HbA1c) was 5.7% (reference: 4.0-6.0%). She had been managed primarily in the oncology setting after being diagnosed with liver metastasis from HR+/HER2- breast cancer 2 years earlier. Her recent anti-tumor treatment regimen included triple therapy with Palbociclib, Toremifene, and Inavolisib (9 mg once daily), which had been initiated shortly before the current hyperglycemic event; Concomitant medications included only vitamin C and compound glycyrrhizin; there was no recent exposure to diabetogenic agents such as systemic corticosteroids, thiazides, or parenteral nutrition. Inavolisib administration was initiated on July 27. On July 29, a fasting blood glucose of more than 12 mmol/L was detected via home monitoring. Lacking severe symptoms, the patient delayed seeking immediate care. However, within just 24 hours (on July 30), she presented to the emergency department with progressive fatigue and a critical blood glucose of 48.0 mmol/L (reference: 3.9–6.1 mmol/L). Her past medical history included chronic liver dysfunction due to metastasis. Prior to Inavolisib initiation in June 2025, the patient exhibited existing liver injury (ALT 87.9 U/L, reference: 7–40 U/L; AST 130.1 U/L,reference: 13–35 U/L; and ALP 603 U/L,reference: <135 U/L.) while maintaining a normal fasting blood glucose of 5.1 mmol/L.

Laboratory results demonstrated severe hyperglycemia with markedly elevated fasting C-peptide and insulin levels, alongside a strictly normal HbA1c and negative diabetes-associated autoantibodies (**Table 1**). Concurrently, profound derangements in liver and biliary function were observed. To comprehensively evaluate other potential precipitants, further clinical workups were conducted. The patient remained afebrile; laboratory panels successfully ruled out acute infectious triggers (normal hemogram) and undisclosed exogenous corticosteroid use (markedly elevated 8 AM endogenous cortisol), with all specific values detailed in **Table 1**. Thyroid ultrasound: a slightly hypoechoic area adjacent to the inferior pole of the left thyroid lobe in the left neck, considering the medical history, tumor metastasis was suspected. Multiple abnormal lymph nodes were found in the left root of the neck and left supraclavicular fossa, suggesting tumor metastasis. Abdominal ultrasound: multiple solid lesions in the liver, considered metastatic carcinoma; peritoneal effusion. Echocardiography: no significant abnormalities in intracardiac structure or blood flow; normal global systolic function of the left ventricle.

**Table 1:** The patient's laboratory test results.

Test item	Test result	Reference range
Diabetes-related indicators		
FPG(mmol/L)	8.6	3.9-6.1
FINS(μU/mL)	41.5	2.6-24.9
FCP(ng/mL)	10.2	1.1-4.4
HbA1c(%)	5.70	4.0-6.0
UKB	–	–
ICA	–	–
GADA	–	–
IAA	–	–
Liver function indicators		
ALT(U/L)	277.5	7-40
AST(U/L)	503.9	13-35
ALP(U/L)	1466	<135
GGT(U/L)	689	7-45
TBIL(μmol/L)	65.4	0-21
DBIL(μmol/L)	57.6	≤8
TBA(μmol/L)	37.6	0-10

FPG, fasting plasma glucose; FINS, fasting insulin; FCP, fasting C-peptide; HbA1c, glycated hemoglobin A1c; UKB, urine ketone bodies; GADA, anti-glutamic acid decarboxylase antibodies; ICA, islet cell antibodies; IAA, insulin autoantibodies; ALT, alanine aminotransferase; AST, aspartate aminotransferase; ALP, alkaline phosphatase; GGT, γ-glutamyl transferase; TBIL, total bilirubin; DBIL, direct bilirubin; TBA, total bile acid.

**Figure 1:**
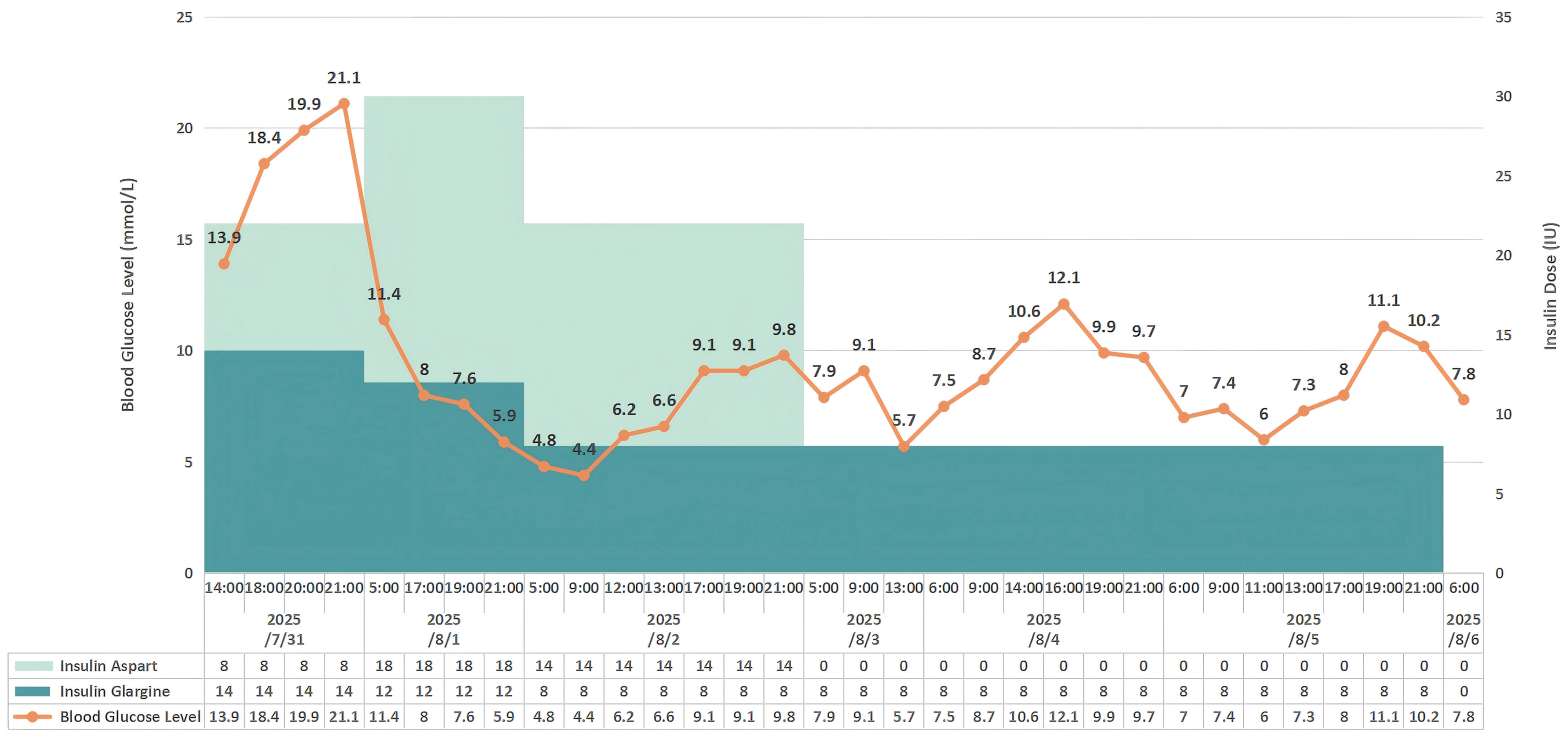
Dynamic changes in blood glucose levels and insulin therapy during hospitalization. The line chart shows serial blood glucose measurements from July 31 to August 6, 2025, together with daily insulin aspart and insulin glargine doses. Blood glucose levels declined rapidly after inavolisib discontinuation and intensive insulin therapy, followed by gradual tapering and complete cessation of insulin within one week.

**Figure 2:**
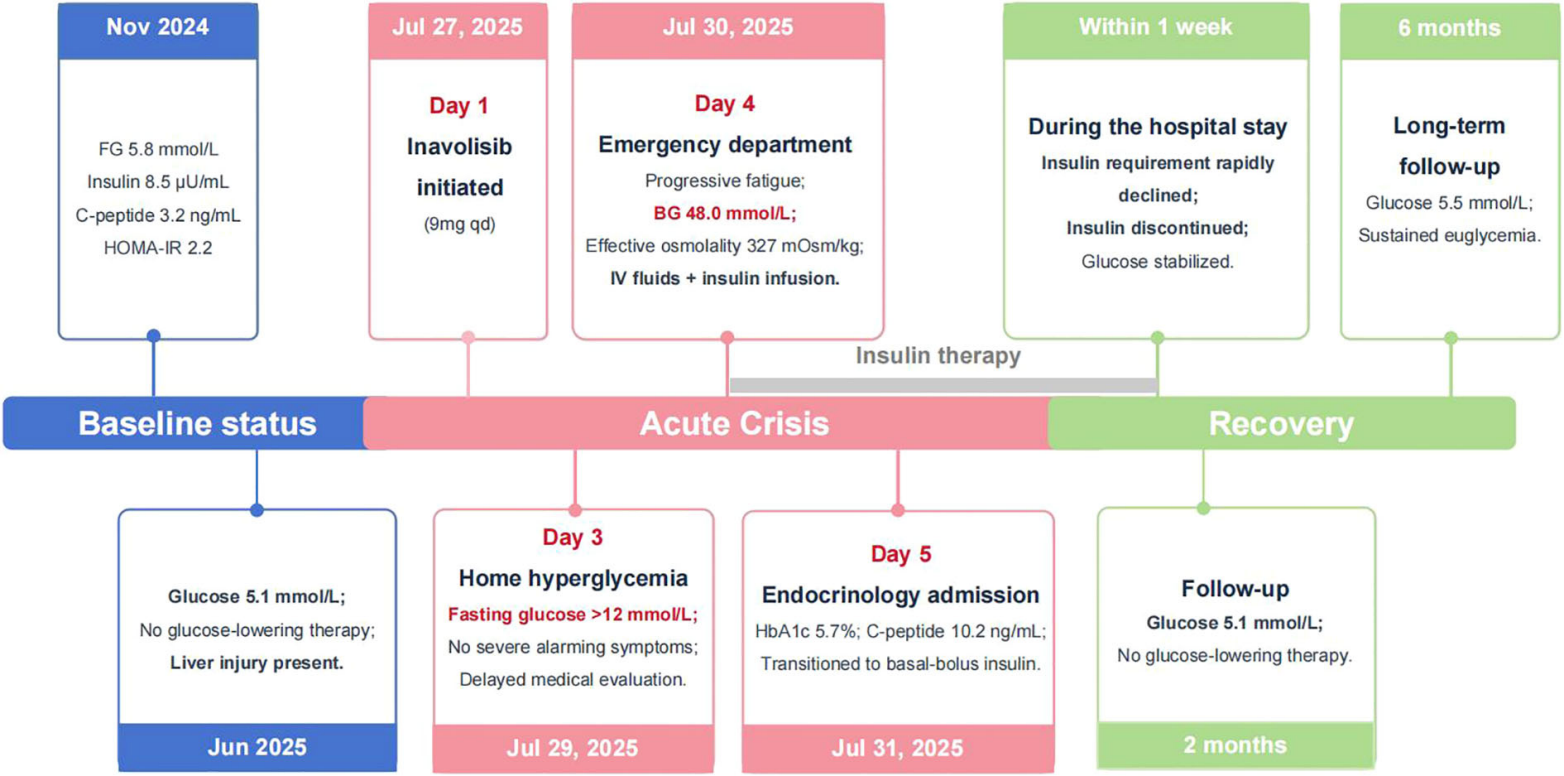
Clinical timeline of inavolisib-induced fulminant-like diabetes and hyperosmolar hyperglycemic state. The timeline summarizes the patient’s baseline metabolic status, initiation of inavolisib, rapid development of severe hyperglycemia and hyperosmolar hyperglycemic state, inpatient insulin-based management, and sustained euglycemia without glucose-lowering therapy during follow-up.

Inavolisib was discontinued while palbociclib and toremifene were maintained. The emergency continuous intravenous insulin pump was subsequently transitioned to an intensive subcutaneous insulin regimen. Specifically, as the patient’s insulin requirement stabilized, the treatment was converted to a basal-bolus regimen combining insulin glargine (initial dose: 14 IU/day) and insulin aspart (initial dose: 8 IU/meal). The total daily insulin requirement rapidly declined from an initial high of 46 IU/day to complete cessation within one week as insulin resistance resolved. The precise dynamic dosing and the corresponding glycemic trajectory are fully detailed in **Figure 1**. Blood glucose was gradually controlled stably. During an outpatient follow-up two months post-discharge, the patient exhibited severe progressive hepatobiliary impairment secondary to tumor progression(AST 178.8 U/L, reference: 13–35 U/L; total bilirubin 310.2 μmol/L, reference: 0–21 μmol/L; and ALP 845.5 U/L, reference: <135 U/L). Her concurrent blood glucose remained normal at 5.1 mmol/L without the need for any glucose-lowering therapy. The most recent follow-up at six months post-discharge confirmed sustained euglycemia (blood glucose 5.5 mmol/L) independent of any antidiabetic medications. The overall episode of care is summarized in **Figure 2**, and key longitudinal metabolic and hepatobiliary findings before admission and during follow-up are summarized in **Table 2**.

**Table 2:** Key longitudinal laboratory findings before, during, and after the hyperglycemic crisis.

Parameter	Reference range	Nov 2024	Jun 2025 (pre-inavolisib)	Jul 30/31, 2025 (crisis/admission)	2 months follow-up	6 months follow-up
Glucose (mmol/L)*	3.9–6.1	5.8	5.1	48.0	5.1	5.5
HbA1c (%)	4.0–6.0	NA	NA	5.7	NA	NA
Fasting insulin (μU/mL)	2.6–24.9	8.5	NA	41.5^#^	NA	NA
C-peptide (ng/mL)	1.1–4.4	3.2	NA	10.2	NA	NA
HOMA-IR	<2.5	2.2	NA	NA	NA	NA
Urine ketone bodies	Negative	NA	NA	Negative	NA	NA
ALT (U/L)	7–40	NA	87.9	277.5	255.9	123.3
AST (U/L)	13–35	NA	130.1	503.9	178.8	357.3
ALP (U/L)	<135	NA	603	1466	845.5	663.9
Total bilirubin (μmol/L)	0–21	NA	13.2	65.4	310.2	112.5
ICA / GADA / IAA	Negative	NA	NA	Negative	NA	NA

NA indicates not reported or not available.

*Pre-treatment values represent fasting glucose when available; the crisis value represents emergency blood glucose at presentation.

#Insulin was measured after emergency insulin infusion and should be interpreted with caution.

## Discussion

This report details a case of rapid-onset hyperglycemia and HHS in a patient with PIK3CA-mutated HR+/HER2- advanced breast cancer receiving Inavolisib therapy. The HR+/HER2- subtype is the most common breast cancer in clinical practice, with approximately 28%–46% of these patients harboring PIK3CA mutations (10). Such mutations activate the PI3K/AKT/mTOR pathway (3, 11), thereby driving tumor growth, promoting drug resistance, and facilitating metastasis (12).

The PI3K pathway is also a key mediator of the glucose-lowering effect of insulin (4). Insulin receptor binding activates PI3Kα downstream, subsequently stimulating tissue glucose uptake, glycolysis, and glycogen synthesis. While Inavolisib, a highly selective inhibitor, exerts its anti-tumor activity by inhibiting this pathway and promoting the degradation of mutant p110α (13–15), this inhibition simultaneously blocks insulin’s metabolic action. This blockage results in peripheral insulin resistance and increased hepatic glucose output, consequently inducing hyperglycemia (6, 8, 16). Oncogenic PIK3CA mutations activate the PI3K/AKT pathway, which theoretically mimics rather than opposes insulin action. Thus, the severe hyperglycemia was exclusively driven by Inavolisib’s pharmacological blockade, not the underlying somatic mutation. Hyperglycemia is thus established as the most common adverse event associated with PI3Kα inhibitors. In a phase 2/3 trial of Inavolisib (6, 8), the incidence of hyperglycemia in patients in the treatment group was 58.6%. Early-phase clinical data have also evaluated inavolisib in combination with palbociclib and endocrine therapy in patients with PIK3CA-mutated HR-positive/HER2-negative advanced or metastatic breast cancer (17). Furthermore, alpelisib, another PIK3CA inhibitor in the same class, demonstrated a comparable metabolic liability in the SOLAR-1 trial, with 63.7% of patients developing hyperglycemia (18, 19). These findings further indicate that PIK3CA inhibitors generally possess the property of inducing hyperglycemia (20–22). When blood glucose levels are significantly elevated (typically > 33.3mmol/L), the resulting glucosuria overwhelms tubular reabsorption, generating a profound osmotic diuretic effect (23). This leads to a massive urinary loss of water and electrolytes (especially sodium). Failure to adequately replete fluids subsequently results in reduced blood volume, diminished effective circulating volume, and pathologically elevated serum osmolarity, precipitating a HHS (24).

The risk of hyperglycemia associated with PI3Kα inhibitors is concentrated in the early stage of treatment, with a median time to onset of approximately 15 days (25, 26). This risk is notably pronounced in patients possessing pre-existing risk factors, including impaired fasting glucose (>7 mmol/L), elevated baseline HbA1c (>5.7%), obesity (BMI ≥30 kg/m²), or long-term excessive carbohydrate consumption (26–28). Consequently, current guideline recommendations necessitate systematic blood glucose-related examinations prior to initiating PI3K inhibitor therapy, followed by rigorous monitoring of glucose changes post-treatment (29, 30). Specifically, it is advised that for the initial 30 days of treatment, both fasting blood glucose and 2-hour postprandial blood glucose levels be measured at least twice per week (29).

This case involves a 59-year-old female patient diagnosed with metastatic breast cancer, who was initiated on combination therapy including Inavolisib, palbociclib, and toremifene. Severe hyperglycemic (blood glucose 48.0 mmol/L) occurred acutely within 72 hours (on Day 3 of treatment) of drug initiation. Concomitant laboratory tests revealed significantly elevated insulin and C-peptide, alongside negative diabetes-related autoantibodies and an absence of ketoacidosis manifestations. Further abdominal ultrasound was performed, which excluded pancreatic disease as the cause of hyperglycemia, showing no pancreatic enlargement, effusion, or space-occupying lesions. Hyperglycemia emerged after Inavolisib initiation and resolved post-discontinuation, with rapid blood glucose reduction thereafter, which further supports a direct association between hyperglycemia and Inavolisib, consistent with a ‘probable’ causality based on the Naranjo algorithm.

In terms of differential diagnosis, several etiologies for acute severe hyperglycemia were systematically evaluated. First, the absence of autoantibodies and markedly elevated C-peptide levels ruled out classic fulminant type 1 diabetes. Second, the strictly normal HbA1c at admission and normal metabolic biochemical parameters prior to treatment excluded an acute presentation of previously undiagnosed type 2 diabetes. Third, the absence of acute infection and the lack of systemic corticosteroid exposure ruled out acute stress hyperglycemia and exogenous steroid-induced diabetes. Fourth, hepatogenous diabetes secondary to her extensive liver metastases was excluded, as her blood glucose remained completely normal during subsequent severe hepatic failure. Fifth, normal abdominal imaging ruled out pancreatogenic diabetes. Finally, other drug-induced possibilities were excluded, as her concurrent targeted therapies lack known associations with profound insulin resistance. Thus, the diagnosis unequivocally points to metabolic toxicity induced by Inavolisib.

From a diagnostic perspective, the patient exhibited a fulminant-like hyperglycemic presentation, characterized by rapid onset, markedly elevated glucose at first presentation, and a normal HbA1c level, resembling but not fulfilling the classic phenotype of fulminant type 1 diabetes (31). Although the measured insulin level was confounded by prior emergency insulin infusion, the concomitant C-peptide level remained informative because exogenous insulin does not contain C-peptide. The preserved C-peptide level, together with negative diabetes-related autoantibodies, argues against acute β-cell failure and instead supports severe drug-induced insulin resistance as the dominant pathophysiological mechanism in this case. Therefore, rather than assigning this presentation to a specific diabetes subtype, we believe it is more appropriate to describe it as an inavolisib-induced fulminant-like hyperglycemic state.

HHS is one of the life-threatening hyperglycemic emergencies in diabetes mellitus, characterized by severe hyperglycemia, hyperosmolarity, profound dehydration, absence of significant ketoacidosis, and variable degrees of neurological impairment (32–35). For the present patient, the blood glucose level of 48 mmol/L, and the effective serum osmolarity was 327 mOsm/kg fulfilled the diagnostic criteria for HHS (23, 34). However, the clinical manifestations deviated significantly from typical HHS. The patient remained conscious with a normal mental status and only reported fatigue. This atypical presentation is likely attributable to the patient receiving initial fluid therapy early in the disease course (34). At that stage, the hyperosmolar environment had not yet caused irreversible damage to the central nervous system, and extracellular fluid dehydration had not progressed to a state of severe decompensation. The standard approach in clinical practice is the combined adoption of aggressive fluid replacement and low-dose insulin therapy for glycemic control (36).

The uniqueness of this case is highlighted by the unusually rapid onset (within 72 hours of drug initiation) and severity (blood glucose 48.0 mmol/L) of hyperglycemia, which exceeds previous reports on PI3Kα inhibitor-related adverse events (25, 26). This severity may be attributed to Inavolisib’s higher selectivity and inhibitory activity against PI3Kα, leading to faster onset and more severe metabolism-related side effects, based on the drug’s mechanism of action (37). Critically, the patient had a normal baseline BMI (19.55 kg/m²) and HbA1c level (5.7%). This demonstrates that even patients with normal baseline metabolic indicators may experience rapid and severe hyperglycemia when using Inavolisib, suggesting susceptibility extends beyond known risk factors (22). Clinicians must recognize that patients without high-risk factors may still experience severe drug-related adverse effects and should strengthen blood glucose monitoring during treatment to promptly identify and manage metabolic abnormalities.

Drawing on the management lessons from this case and existing literature, we propose the following clinical recommendations for blood glucose management during Inavolisib treatment (16). First, we emphasize the critical role of proactive lifestyle interventions and risk stratification prior to initiating Inavolisib to avoid or mitigate the incidence of hyperglycemia. Specifically, patients should receive pre-treatment dietary counseling focusing on a low-carbohydrate diet and be encouraged to engage in regular physical activity (4, 16, 29). All patients scheduled to receive Inavolisib should undergo routine screening of fasting blood glucose and HbA1c before treatment to establish the baseline metabolic status. Following treatment initiation, high-frequency blood glucose monitoring should be implemented, such as daily monitoring of fasting and postprandial blood glucose in the first 2 weeks, with subsequent frequency adjustment based on glucose stability (29). This intensive monitoring is necessary because pharmacokinetically, Inavolisib exhibits rapid absorption with a median time to peak plasma concentration (*T_max_*) of approximately 3 hours and a mean terminal half-life (*t_1/2_*) of 16.4 hours, enabling it to reach steady-state concentrations within the first week of treatment (15, 32, 37). While the median onset time for hyperglycemia with other PI3Kα inhibitors like alpelisib is 15 days, clinical data indicate that inavolisib induces hyperglycemia earlier, with a median onset of 7 days (8, 26). Considering the *T_max_* of 3 hours and median onset time of 7 days, standard twice-weekly monitoring creates three- to four- day blind spots where acute fasting or postprandial glucose spikes may go undetected. Consequently, daily monitoring during the initial two weeks is required to capture these rapid metabolic shifts and prevent severe hyperglycemic emergencies. In this case, the lack of close monitoring during the initial stage of medication led to blood glucose progressing to a critical value of 48.0 mmol/L, highlighting that earlier, intensified monitoring may enable timely intervention and prevent the occurrence of critical conditions. Furthermore, patients presenting with symptoms related to elevated blood glucose, including thirst, polyuria, or fatigue, must be instructed to seek medical attention immediately for urgent evaluation.

Second, the management of inavolisib-related hyperglycemia should be individualized according to the severity and tempo of glucose elevation (4). In our case, the drug-induced dysglycemia developed abruptly and progressed rapidly, but the metabolic disturbance was transient and responded promptly to insulin-based therapy after inavolisib discontinuation. Therefore, for patients presenting with marked hyperglycemia, rapid glucose escalation, or hyperglycemic emergencies such as HHS, insulin-based therapy should remain the first-line approach for prompt metabolic control, together with temporary interruption of inavolisib when clinically indicated (4, 38). By contrast, oral glucose-lowering agents may not be necessary in every case. For selected patients with milder hyperglycemia, stable clinical status, and the ability to undergo close follow-up, insulin-sensitizing agents such as metformin may be considered on an individualized basis (4, 16). Additionally, future clinical trials are needed to evaluate whether prophylactic administration of insulin-sensitizing agents in high-risk patients can effectively prevent the onset of PI3Kα inhibitor-induced diabetes (29, 30). As treatment-related dysglycemia has increasingly been recognized across anticancer therapies, careful baseline metabolic assessment, patient education, close glucose monitoring, and early multidisciplinary collaboration are essential to reduce preventable severe events and to optimize both oncologic and metabolic outcomes (16, 29, 30, 39).

The necessity for temporary Inavolisib discontinuation due to severe hyperglycemia underscores the critical challenge in the management of PI3Kα inhibitor-associated toxicity. Although existing data form related agents suggest that low-dose strategies may mitigate hyperglycemia while partially preserving anti-tumor efficacy (40), the specific clinical implications for Inavolisib remain poorly defined. Critically, there is a significant paucity of evidence from prospective trials or real-world studies to confirm whether dose adjustment can effectively manage hyperglycemia or more fundamentally, whether it compromises antitumor efficacy in PIK3CA-mutated breast cancer. This evidentiary gap severely restricts clinicians’ ability to guide informed dose modification, thereby impeding the development of personalized regimens that optimally balance efficacy and tolerability.

## Patient perspective

Upon admission, the patient recalled her initial reaction to the sudden hyperglycemia. She had noticed a home blood glucose reading above 12 mmol/L but did not grasp the severity of the situation. Having no prior history of diabetes, she simply assumed the high number was a temporary spike from her diet. She felt unusually tired, but since she lacked classic diabetic symptoms like excessive thirst, frequent urination, or increased appetite, a medical emergency never crossed her mind. It was only when her fatigue became overwhelming that she realized something was fundamentally wrong and sought urgent care. Once the intensive insulin therapy stabilized her blood glucose, she expressed a profound sense of relief. Her physical exhaustion lifted, and her intense anxiety regarding the sudden crisis resolved, leaving her highly appreciative of the prompt medical intervention.

## Conclusion

This case highlights that inavolisib can precipitate severe hyperglycemia within days of treatment initiation and may rapidly progress to HHS, even in patients without known dysglycemia. A key clinical message is that normal baseline glycemic indices do not reliably exclude the risk of severe toxicity. Careful metabolic risk assessment before treatment, close glucose monitoring during the early treatment phase, prompt interruption of inavolisib when clinically indicated, and rapid insulin-based management for severe presentations are essential to facilitate early recognition and prevent hyperglycemic emergencies in clinical practice.

## Data Availability

The original contributions presented in the study are included in the article/supplementary material. Further inquiries can be directed to the corresponding author.
